# Diversity of the Formicidae (Hymenoptera) carrion communities in Lisbon (Portugal): preliminary approach as seasonal and geographic indicators

**DOI:** 10.1080/20961790.2017.1372875

**Published:** 2017-09-22

**Authors:** António Neto-Silva, Ricardo Jorge Dinis-Oliveira, Catarina Prado e Castro

**Affiliations:** a Department of Public Health, Forensic Sciences and Medical Education, Faculty of Medicine, University of Porto, Porto, Portugal; b Department of Sciences, IINFACTS – Institute of Research and Advanced Training in Health Sciences and Technologies, University Institute of Health Sciences (IUCS), CESPU, CRL, Gandra, Portugal; c UCIBIO, REQUIMTE – Laboratory of Toxicology, Department of Biological Sciences, Faculty of Pharmacy, University of Porto, Porto, Portugal; d Department of Life Sciences, Centre for Functional Ecology, University of Coimbra, Coimbra, Portugal

**Keywords:** Forensic science, forensic entomology, Formicidae, sarcosaprophagous community, decomposition process, postmortem interval, seasonal study

## Abstract

The value of the Formicidae (Insecta, Hymenoptera) community in forensic investigations is poorly studied in Portugal. In order to better understand the structure and dynamics of this group of insects in cadavers and their putative value in forensic investigations, studies were carried out in Lisbon area during one year. Piglet carcasses were used as a model of human decomposition. The entomofauna attracted to the carcasses was collected over a period of about 2 months in each season of the year. The collection of ants was performed at regular intervals, daily in the first 23 days and then with intervals of 2, 3 or 5 days until the end of the experiment. Five stages of cadaveric decomposition were recognized with the ants being present in all of them. Three hundred and nine specimens were captured: 7 in autumn, 6 in winter, 90 in spring and 206 in summer. These specimens belong to three subfamilies and seven different species. Four of them had never been mentioned before as being associated to cadavers in this geographical area. Spring and summer had the greater diversity of species and a larger number of individuals. *Temnothorax luteus* was the dominant species in spring and *Tapinoma nigerrimum* in summer. Corroborating the information of other similar studies carried out in the Iberian Peninsula, the sarcosaprophagous Formicidae community found in Lisbon is unique and different from other studied locations, which supports the need to perform regional studies. Our results shown that ants do not present a definite pattern of succession, but some species have the potential to be seasonal or geographic indicators.

## Introduction

The entomofauna present at a crime scene represents an important evidence about the place and the time of death, frequently assisting in the calculation of the postmortem interval (PMI) [1–3]. Forensic entomology uses arthropods, mainly from the class Insecta, in criminal investigations. It applies to several areas, namely to medical–legal cases, urban and stored products infestations [1,3,4]. The medical–legal branch of forensic entomology focuses on arthropods that are found or infest human corpses. These colonizers can be used in various ways in the investigation: to estimate the PMI through the study of their life cycle or succession patterns of colonization; to know if the body was moved; or to associate suspects with the crime scene [1,5]. Insects are an important tool in solving crimes and in recent years forensic entomology has been evolved immensely with recent and innovative studies [6–13]. A corpse is visited by a wide variety of arthropods called sarcosaprophagous which represent a community that is specific from a location and depends on its soil and climate conditions [14]. The in-depth knowledge of this community in a particular region is an asset for criminal investigation.

While forensic entomology has been progressing internationally, in the Iberian Peninsula, there is still little research in this area [6,15–17] and the studies are even more scarce in Portugal, focusing mainly on the study of flies (Diptera) and beetles (Coleoptera) on corpses [18–21]. Although ants (Formicidae) are one of the most abundant groups of the sarcosaprophagous community, they are poorly studied in the forensic context [22], even though some works were carried in the Iberian Peninsula [23–26].

According to the categories described by Smith [3], arthropods can be: necrophagous – when feeding exclusively on parts of the corpse; predators and parasites of necrophagous species; omnivores – that feed on both the corpse and the species that visit it; and adventive – species that appear randomly because the corpse is in its habitat. In forensic context, the ants, due to their eating habits, are considered omnivores. They are almost ubiquitous and have different eating habits, some are seed collectors, others are predators and some create symbioses with other insects to obtain food. Ants that feed on carcasses can feed on the carcass itself, or the fauna associated with it [3,12,27,28]. Several experimental studies on animal carcasses have confirmed the presence of ants in the different stages of cadaveric decomposition, being mainly opportunistic predators of eggs and larvae of other insects [2,10,13,26,29]. Even though observed in the different stages of decomposition, the value of ants is often neglected by forensic pathologists and investigators, and its effect on cadaverous remains is not much appreciated, as it can bring confusion to research. They are able to remove eggs, larvae and even adults of Diptera and Coleoptera [2,3,6,10,27], thus affecting the normal rate of decomposition and interfering in the estimation of the PMI based on the succession of insects [22,30,31]. In addition, some species feed on the corpse and induce artefacts, often confused with *antemortem* or *perimortem* wounds. An experienced pathologist can distinguish it, but, in certain circumstances, they are difficult to differentiate. Therefore, it is necessary to take into account the size and orientation of the lesions and habitat where the remains were found and try to find evidence of ant activity that could support the final diagnosis [7,22,28,32,33]. Another intrusive behaviour of the ants is to monopolize exposed wounds and natural orifices of cadavers burying them with soil particles. Ants of the species *Solenopsis invicta* (Buren) have been documented to have this behaviour that hinders the access of other insects to the most susceptible parts of colonization of cadavers, thus affecting the decomposition process [34]. However, the effects of ants in these situations are dependent on species, abundance and their geographical area of distribution [26]. In many cases, ants are the group of arthropods numerically dominant on the corpse, since they are social insects that live in colonies that can reach thousands of individuals (queen, soldiers and workers). Some species, such as *Anoplolepsis longipes* (Jerdon), have already been used to estimate the PMI, taking into account the minimum time this species needs to establish a colony [35].

This study aims to investigate the composition and dynamics of the Formicidae community in cadavers during the different seasons of the year, in Lisbon (Portugal), in an attempt to determine its utility as forensic indicators. The information on the species found will allow us to increase the knowledge about the group in this geographical area and in the Iberian Peninsula, by comparing the results found in other similar studies.

## Materials and methods

### Carcasses and experimental procedures

The experiments were carried out from October 2006 to August 2007 in Lisbon. Accordingly to Köppen–Geiger climate classification system, the region is classified as type Csa; temperate Mediterranean climate with hot summers and rainy winters [36]. Also, accordingly to data from the Portuguese national weather service (IPMA – Instituto Português do Mar e da Atmosfera), the average climatological values of precipitation and temperature of the seasons are: in spring 119.5 mm, 12.6–14.1 °C; in summer 11.4 mm, 20.2–22.1 °C; in autumn 108.5 mm, 15.6–17.0 °C and in winter 192.4 mm, 9.5–10.8 °C. The study was performed at Agronomy Higher Institute (Tapada da Ajuda – 38°42′41″N, 09°11′28.6″W), a forested area within the urban perimeter composed mainly of *Ailanthus altissima* (Mill) Swingle, *Fraxinus angustifolia* (Vahl) and *Ulmus minor* (Mill). The study site was in the shade and away from anthropological activities.

**Figure 1.**
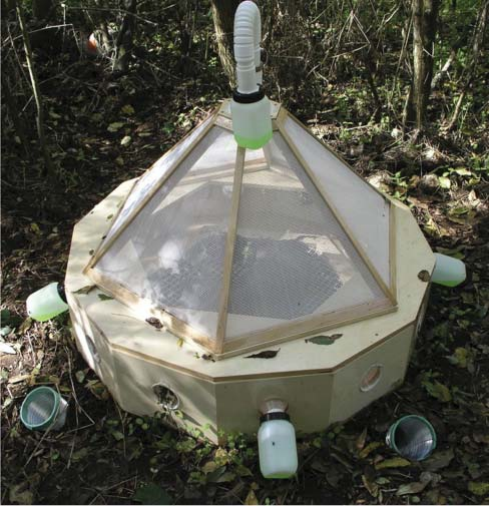
Modified Schoenly trap.

Aiming to adjust the four seasons of the year, four experiments were carried out with duration of 8–10 weeks each. The dates of the experiments were the following: autumn between October 18 2006 and January 2 2007; winter between January 17 2007 and April 3 2007; spring between April 16 2007 and June 16 2007; and summer between June 27 2007 and August 27 2007. For each experiment, one piglet, *Sus scrofa domesticus* (Linnaeus), weighing between 7.5 and 8 kg was sacrificed through an incision in the jugular vein as defined by the veterinary. All sacrifice-related procedures were performed as to give the proper animal care, to reduce suffering and stress. Experimental animal procedures were in agreement with the European Council Directive (2010/63/EU) guidelines that were transposed into Portuguese law (Decree-Law no. 113/2013, August 7th) and complied with the guidelines of the committee and National Council of Ethics for the Life Sciences (CNECV). The piglet carcass was then placed inside a modified Schoenly trap acting as bait [37]. This trap (Figure 1) allows the entomofauna to be continuously collected, while allowing the cadaveric decomposition process to occur naturally. The bottles in the trap from which the arthropods were collected contained a 40% solution of ethylene glycol with formalin and detergent, which allowed them to be killed and stored temporarily. The collection and replacement of the capture bottles was performed daily in the first 23 days and then every 2, 3 and 5 days until the end of the experiment.

The temperature was recorded hourly within the trap with a HOBO® data logger. Day 1 corresponds to the date of death and placement of the piglet into the trap. The collected specimens were stored in 80% ethanol and deposited in the collection of the Department of Animal Biology, University of Lisbon.

All collected ants were identified to the species level. For this task, a binocular stereomicroscope and dichotomous keys [38] related to this group of insects were used. Biodiversity parameters were calculated, namely abundance and specific richness for each season of the year and for each stage of decomposition.

### Decomposition process

The decomposition stages of the piglets were evaluated according to Anderson and VanLaerhoven [39]. Briefly, the stages were described as: Fresh (F), fresh appearance without odour; Bloated (B), bloating, initiating as slight inflation of the abdomen with odour of putrefaction; Decay (D), the carcass starts to deflate, larval masses feeding on soft tissues with strong odour of decay; Advanced decay (AD), intense migration of larvae with decrease of the odour, most of the flesh has been removed at the end of this stage; and Dry (DR), carcass consists of bones, skin and hair with little to no odour.

## Results

**Figure 2.**
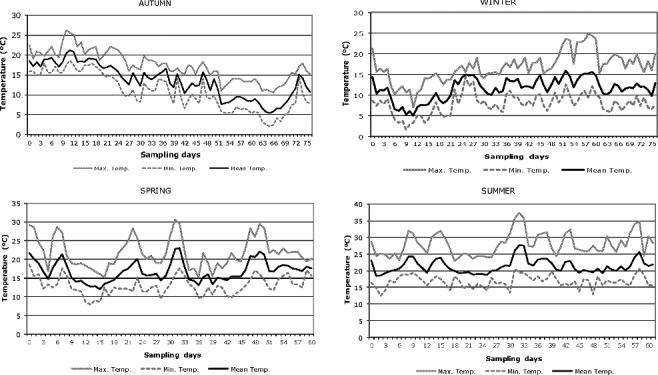
Temperature data for the studied periods.

The variation of the temperatures in the sampling periods of each season is represented graphically in Figure 2. Taking into account the first two weeks of carcass exposure, the mean temperature was (18.7 ± 1.3) °C in autumn, (8.4 ± 2.7) °C in winter, (16.8 ± 3.1) °C in spring and (21.2 ± 2.0) °C in summer.

In this study, 309 specimens of ants were collected, 7 in autumn, 6 in winter, 90 in spring and 206 in summer. These specimens belong to three subfamilies and seven different species, as shown in Table 1. All the decomposition stages were observed but with different durations in the different seasons of the year, with the exception of the autumn in which stage D was not observed since deflation started simultaneously with larval migration. Also in autumn, differently from the other seasons, the humidity and mould were usually present in the DR stage.

**Table 1.** Absolute seasonal abundance and species richness [*n*(%)].

			Seasons				
Family	Subfamily	Species	Autumn	Winter	Spring	Summer	Total (*n*)
Formicidae	Dolichoderinae	*Tapinoma nigerrimum*	–	–	–	109(53%)	109
	Formicinae	*Plagiolepis pygmaea*	–	–	7(8%)	23(11%)	30
		*Aphaenogaster senilis*	–	5(83%)	7(8%)	7(3%)	19
		*Crematogaster auberti*	–	–	3(3%)	13(6%)	16
	Myrmicinae	*Crematogaster scutellaris*	–	–	8(9%)	27(13%)	35
		*Temnothorax luteus*	4(57%)	1(17%)	58(64%)	26(13%)	89
		*Tetramorium semilaeve*	3(43%)	–	7(8%)	1(1%)	11
Abundance			7	6	90	206	309
Species richness			2	2	6	7	–

–: not obtained.

Although with different abundances and different species, the Formicidae family was present in all stages of decomposition of the piglet carcasses (Tables 2–5) in the four seasons of the year.

**Table 2.** Species found in autumn, day and stage of decomposition (*n*).

	F			B				AD																	DR																
Species	1	2	3	4	5	6	7	8	9	10	11	12	13	14	15	16	17	18	19	20	21	22	23	25	27	29	31	33	35	37	40	43	46	49	52	55	58	61	66	71	76
*Temnothorax luteus*										1													2	1																	
*Tetramorium semilaeve*	1											1												1																	

F: Fresh, fresh appearance without odour; B: Bloated, bloating, initiating as slight inflation of the abdomen with odour of putrefaction; AD: Advanced decay, intense migration of larvae with decrease of the odour, most of the flesh has been removed at the end of this stage; DR: Dry, carcass consists of bones, skin and hair with little to no odour.

**Table 3.** Species found in winter, day and stage of decomposition (*n*).

	F																	B								D			AD							DR					
Species	1	2	3	4	5	6	7	8	9	10	11	12	13	14	15	16	17	18	19	20	21	22	23	25	27	29	31	33	35	37	40	43	46	49	52	55	58	61	66	71	76
*Aphaenogaster senilis*																																	4					1			
*Temnothorax luteus*																																			1						

F: Fresh, fresh appearance without odour; B: Bloated, bloating, initiating as slight inflation of the abdomen with odour of putrefaction; D: Decay, the carcass starts to deflate, larval masses feeding on soft tissues with strong odour of decay; AD: Advanced decay, intense migration of larvae with decrease of the odour, most of the flesh has been removed at the end of this stage; DR: Dry, carcass consists of bones, skin and hair with little to no odour.

**Table 4.** Species found in spring, day and stage of decomposition (*n*).

	F			B			D	AD												DR																		
Species	1	2	3	4	5	6	7	8	9	10	11	12	13	14	15	16	17	18	19	20	21	22	23	25	27	29	31	33	35	37	40	43	46	49	52	55	58	61
*Aphaenogaster senilis*							2	1													1	1											1	1				
*Crematogaster auberti*																				3																		
*Crematogaster scutellaris*																				5							1							1			1	
*Plagiolepis pygmaea*								1	1									1								1		1						1		1		
*Temnothorax luteus*	2	8				3	6	2		2		2						1		4		2	1				1	4			2	3	3	4	6		2	
*Tetramorium semilaeve*							1						1										1				1						1		1	1		

F: Fresh, fresh appearance without odour; B: Bloated, bloating, initiating as slight inflation of the abdomen with odour of putrefaction; D: Decay, the carcass starts to deflate, larval masses feeding on soft tissues with strong odour of decay; AD: Advanced decay, intense migration of larvae with decrease of the odour, most of the flesh has been removed at the end of this stage; DR: Dry, carcass consists of bones, skin and hair with little to no odour.

**Table 5.** Species found in summer, day and stage of decomposition (*n*).

	F		B			D	AD						DR																									
Species	1	2	3	4	5	6	7	8	9	10	11	12	13	14	15	16	17	18	19	20	21	22	23	25	27	29	31	33	35	37	40	43	46	49	52	55	58	61
*Aphaenogaster senilis*	1		1	1						1		1				1			1																			
*Crematogaster auberti*						3	5	5																														
*Crematogaster scutellaris*	13	3		4	1		1					1	1		1		1																					1
*Plagiolepis pygmaea*	1	1			2						1		1		1	1	1		1					1			1	3				3			1	4		
*Tapinoma nigerrimum*																	19	5	11	5	2	7	3	3	9	12	9	10	3	6	1	4						
*Temnothorax luteus*	2	2		3	1					2				1	1	1	1		2		1		1					4	1			1	2					
*Tetramorium semilaeve*																		1																				

F: Fresh, fresh appearance without odour; B: Bloated, bloating, initiating as slight inflation of the abdomen with odour of putrefaction; D: Decay, the carcass starts to deflate, larval masses feeding on soft tissues with strong odour of decay; AD: Advanced decay, intense migration of larvae with decrease of the odour, most of the flesh has been removed at the end of this stage; DR: Dry, carcass consists of bones, skin and hair with little to no odour.

Autumn and winter were the seasons with lower specific richness and lower abundance of formicides that visited the carcasses. In autumn, only two species appeared: *Tetramorium semilaeve* that was present in the F and AD stages; and *Temnothorax luteus* that was present in the AD stage (Table 2). In winter, two species were also captured: *Aphaenogaster senilis* in the AD and DR stages; and *T. luteus* represented only by one individual in the AD stage (Table 3).

Spring and summer were the seasons that presented the higher specific richness and abundance. In spring, there were six different species: *T. luteus* was present in all stages of cadaveric decomposition; *T*. *semilaeve* and *A. senilis* were found in stages D, AD and DR; *Plagiolepis pygmaea* appeared in the AD and DR stages; and *Crematogaster auberti* and *Crematogaster scutellaris* were only captured in the DR stage. The most abundant species during spring was *T. luteus* representing 64% of all individuals collected. Summer was the season with the highest number of captured ants (206 specimens) and also with the highest specific richness, with seven different species captured: *A. senilis*, *C. scutellaris*, *P. pygmaea* and *T. luteus* which were present in all states of cadaveric decomposition with the exception of stage D; the species *C. auberti* was present in stages D and AD; and *Tapinoma nigerrimum* appeared exclusively in this season of the year and was the most abundant species with 53% of relative species abundance; nevertheless, it only appeared in the DR stage of decomposition.

*T. luteus* was the only species that was present in the four seasons, being more abundant during the spring and summer (Table 1). *A. senilis* and *T. semilaeve* were present in three seasons, but the number of specimens collected was very low. *P. pygmaea* and the species of the genus *Crematogaster* were only observed in spring and summer. The only species that was found in just one season was the *T. nigerrimum* and it was in the summer (Table 1). In autumn and winter, the two species found were more abundant in the AD stage. In spring, in the DR stage, we observed the highest specific richness with the presence of the six species. In the summer, it was also the DR stage that presented the highest specific richness with six species.

## Discussion

The information from carrion entomofauna is particularly useful in the calculation of the PMI [1–3,35]. However, knowledge about several groups of arthropods belonging to this community, such as the Formicidae, is scarce. This family represents an important part of the sarcosaprophagous community and needs to be studied in greater depth in order to overcome the lack of knowledge about this group of insects in forensic context. The environmental conditions of spring and summer regarding temperature, humidity and food availability explain the greater diversity of ant species and a larger number of individuals present in the environment [3]. Indeed, these seasons presented the greater specific richness and abundance (Table 1). Of the seven species found, four have already been mentioned several times in studies performed on cadavers (*T. nigerrimum*, *C. scutellaris*, *P. pygmaea* and *T. semilaeve*).

*T. nigerrimum* (Nylander) is a polymorphic species, with individuals ranging from 3.2 to 5.2 mm length, characterized by their uniform black colour. Specimens usually form columns of mass workers during foraging. They are active at moderate temperatures and with high relative humidity. Without these conditions, they are more easily found in the shade or in night activities [40]. It is omnivorous, generalist and opportunistic [41], can feed on aphid exudates and may also be predator of larvae, pupae and adults of other insects [42]. The species is distributed throughout the Mediterranean and very frequent in the centre and south of the Iberian Peninsula [38], being very resistant to human activities. *T. nigerrimum* is known for causing skin lesions or modifying existing wounds in bodies by feeding itself, leading to misinterpretations by forensic pathologists [28,43]. In addition, this species should be considered as an opportunistic member of the sarcosaphagous community since it is able to remove eggs and larvae of dipterans, which may slow the decomposition process and make an incorrect assessment of the PMI [22,28]. In this study, this species was only found in the summer, and therefore could be a potential seasonal indicator, was present exclusively at the DR decomposition stage and was the most abundant species in this season (Tables 1–5). Knowing the seasonal activity of this species, it was predictable that in the summer, it would be particularly active because at this time of the year, temperatures are more favourable to its forage activity [41]. They were captured in large numbers when compared to the other species, probably due to the mass forage activity of workers, characteristic of this species [40].

Regarding *C. scutellaris* (Olivier) and *C. auberti* (Emery), the ants of this genus are unmistakable due to individual features, namely the heart-shaped abdomen and the dorsal joining of the post-petiole to the abdomen. The ants usually present approximately 4 mm length, and the distinction of *C. scutellaris* from the species *C. auberti* is based on the colouration; *C. scutellaris* has the head and part of the abdomen red and *C. auberti* is uncoloured brown [38]. Both species are omnivorous, feed on aphid exudates and predate eggs [44], larvae and other insects [45]. These ants are distributed throughout the Mediterranean region, very abundant in the centre, south and coast of the Iberian Peninsula [38]. The *Crematogaster* species were present in spring and summer. In the spring, the two species only appeared in the DR stage. In the summer, *C. auberti* were collected in the D and DR stages while *C. scutellaris* appeared in the different stages, with the exception of D, presenting in large numbers at the beginning of stage F. The presence of this genus in spring and in the DR stage can be explained by the existence of some larval activity, pupae and adults of certain groups of dipterans (e.g. Piophilidae) which would attract it to the carcass remains [21]. The availability of food provided by the carcass, both in stage F (skin and carcass flesh) and in stages D and AD (large numbers of eggs and Diptera larvae) can justify the presence of this genus in summer. *C. scutellaris*, as the *T. nigerrimum*, has already been mentioned as important in the forensic context, since it feeds on corpses, causing postmortem lesions that can be misinterpreted with antemortem and perimortem lesions and also as an active predator of eggs and larvae thus influencing the estimation of the PMI [22,46].

*T. semilaeve* (André) represent small ants of about 2.5–3 mm, light yellow, slightly darker on the head and back of the thorax. The species is monomorphic with striated and rough tegument. It has preference for hot and dry open areas, also occurring in forest edges, urban areas and enclosed spaces. These ants are omnivores, being aggressive and with great resource-dominating capacity [47]. They are distributed throughout the Mediterranean region with a presence throughout the Iberian Peninsula. In our experiment, *T. semilaeve* appeared in all seasons, except in winter, practically in all stages of decomposition, but always in very small numbers.

*P. pygmaea* (Latreille) ants are unmistakable with other genera in the Iberian Peninsula by having 11 segments in the antennas. They are very small with a length of less than 3 mm and brown in colour. They can live among putrefied wood, under rocks and in wooded areas in underground colonies. This species presents several queens per colony forming a network between the nests of the different queens known as polydomous colony. This species is an omnivorous species that feeds on sugary exudates as well as on eggs and larvae from other insects and also from meat and fruit remains [48]. It occurs throughout the Southern part of Europe, being common in the Iberian Peninsula. *P. pygmaea* was only captured in the spring and summer, being more abundant during the summer in the DR stage. These two species are very common in the Iberian Peninsula and have already been reported in other studies of Iberian sarcosaprophagous fauna [49]. The cause of their presence is probably due to its opportunistic habits of obtaining food, being the carcass and its entomofauna attractive for these two omnivorous species [48,47]. Like the previous species, *A. senilis* also appeared in very small numbers, in winter, spring and summer and in several stages of decomposition. The individual way the workers of this species carry their forage activity [50] may explain this reduced number of collected specimens.

*A. senilis* (Mayr) ants present matte appearance, covered by white and thick hairiness with a length of 5.5–7 mm. They are observed in open sunny places, forest edges, fields with shrubs and urban areas. Colonies often move their nests to open areas in the spring and autumn and to shady areas in the summer. Workers are individual foragers moving quickly [50]. They are omnivores feeding on both seeds and animal remains. The species is very common in the Iberian Peninsula [38]. Although the genus *Aphaenogaster* has already been cited as belonging to the sarcosaphagous community [25], *A. senilis* is described for the first time as part of this community.

The classification of ants of the genus *Temnothorax* (Mayr) has undergone several modifications mainly due to morphological variations caused by different temperatures during the pupal stage and by the different types of diet. These different conditions originate in several cases of hybridizations, which make the taxonomy of this group quite complex [51,52]. Workers are usually small (<3 mm). Colonies are typically monogynous with less than 100 workers. They may be arboreal living within hollow stems, old beetle or termite galleries. *Temnothorax* species appear to be trophic generalists, feeding on a wide variety of scavenged items [53]. They are found in the Western Mediterranean region and are considered a cryptic species of *Temnothorax racovitzai* (Bondroit) with which it is often confused taxonomically [52]. In our study, *T. luteus* was the only species captured in all seasons. It belongs to a genus that is currently under review and has little information available. Although the genus has already been cited in Iberian studies of sarcosaprophagous fauna [49], this species is mentioned for the first time in this context. *T. luteus* was also the most abundant species in the spring, being found in all stages of decomposition.

Differently to several families belonging to Diptera and Coleoptera orders, which present definite abundance peaks, respectively, at the beginning and in advanced stages of the decomposition process [20,21], in this study, the ants were found, without a concrete pattern, throughout the whole stages (Tables 2–5). The absence of a consistent pattern of appearance can be explained by the opportunistic feeding habit of the species found [3,24]. Our results indicate that ants do not present an entomological succession pattern. Thus, they cannot be used directly in PMI estimations as many flies and beetles can.

Comparing the species of ants found in Lisbon with the species found in other studies carried out so far in the Iberian Peninsula (Table 6 modified from [26]), we observed that four species (*A. senilis*, *C. auberti*, *T. nigerrimum* and *T. luteus*) had never been mentioned before with forensic interest in this geographical area. On the other hand, there are three species that are common with the communities of the other studies. Indeed, *C. scutellaris* also belongs to the community of Huesca; *P. pygmaea* was also found in Coimbra and Murcia; and the communities of Lisbon, Coimbra and Huesca shared the species *T. semilaeve*. It was possible to verify that the Formicidae community from Lisbon is unique and different from the other areas considered, thus corroborating that the communities of sarcosaprophagous ants can provide a potential application in forensic practice as a geographic indicator [26,49] and emphasizing the importance of this type of studies.

**Table 6.** Formicidae species found in four different studies in the Iberian Peninsula.

Species	Lisbon	Coimbra	Huesca	Murcia
*Aphaenogaster iberica*				×
*Aphaenogaster senilis*	×			
*Camponotus aethiops*			×	×
*Camponotus sylvaticus*				×
*Cataglyphis ibericus*				
*Crematogaster auberti*	×			
*Crematogaster scutelaris*	×		×	
*Formica rufibarbis*			×	
*Lasius grandis*			×	
*Lasius niger*				×
*Linepithema humile*		×		×
*Messor barbarous*				×
*Myrmica specioides*			×	
*Pheidole pallidula*			×	×
*Plagiolepis pygmaea*	×	×		×
*Plagiolepis schmitzii*				×
*Plagiolepis xene*				×
*Ponera coarctara*		×		
*Pyramica membranifera*				×
*Solenopsis* sp.				×
*Tapinoma nigerrimum*	×			
*Temnothorax luteus*	×			
*Temnothorax nylanderi*		×		
*Temnothorax recedens*		×		
*Tetramorium semilaeve*	×	×	×	

Note: The shaded area indicates the common species between this work and the other studies [26].

Regarding the global distribution, the number of ant species decreases with increasing latitudes, altitudes and aridity [54]. Therefore, in comparison to our results (Table 1), it is predictable that, in tropical areas, the specific richness of ants found in cadavers is superior [10,12,22] and that they have more impact on carrion [22]. In addition to the specific richness, the voracity of the species found is also an important point to take into account, as voracious species will have a greater impact on the entomofauna from which they feed and hence in the cadaveric decomposition. The mass forage activity of workers of some species is another important issue because large number causes bigger impact in other insects or in the carcass itself. In this study, the presence in summer of the species *T. nigerrimum* was enough to increase considerably the number of individuals present in the carcass, emphasizing that the effect that ants may have on carrion is mainly dependent on species and its amount or abundance [22,39,55].

## Conclusion

This study is a first approach to the seasonal dynamics of the Formicidae family as a part of the sarcosaprophagous community in Lisbon. It is advisable to carry out more studies, namely by including more samples per season and at several points in the same region in order to carry out more complex statistical calculations that improve the credibility to the extrapolation for humans. This study confirms the forensic importance of the ants, since they interfere with the cadaveric fauna [22,28,30,31,46] and may be useful as geographic and seasonal indicators. None of the species found proved but to be useful for the direct calculation of PMI, since they do not present a definite pattern of succession. Among the species found, none was endemic or of limited geographical distribution, which would be interesting and of great value for forensic investigation, since it would, for example, allow to detect movements of the body postmortem.
